# Impact of Artemisinin-Based Combination Therapy and Insecticide-Treated Nets on Malaria Burden in Zanzibar

**DOI:** 10.1371/journal.pmed.0040309

**Published:** 2007-11-06

**Authors:** Achuyt Bhattarai, Abdullah S Ali, S. Patrick Kachur, Andreas Mårtensson, Ali K Abbas, Rashid Khatib, Abdul-wahiyd Al-mafazy, Mahdi Ramsan, Guida Rotllant, Jan F Gerstenmaier, Fabrizio Molteni, Salim Abdulla, Scott M Montgomery, Akira Kaneko, Anders Björkman

**Affiliations:** 1 Infectious Diseases Unit, Department of Medicine, Karolinska Institutet, Stockholm, Sweden; 2 Zanzibar Malaria Control Programme, Zanzibar, Tanzania; 3 Malaria Branch, Centers for Disease Control and Prevention, Atlanta, Georgia, United States of America; 4 Ifakara Health Research and Development Centre, Dar-es-Salaam, Tanzania; 5 Emergency Medicine Unit, Department of Medicine, Kullbergska Hospital, Katrineholm, Sweden; 6 International Development Group, Research Triangle Institute, Zanzibar, Tanzania; 7 Department of Accident & Emergency, Southern General Hospital, Glasgow, United Kingdom; 8 Italian Co-operation, Dar-es-Salaam, Tanzania; 9 Clinical Epidemiology Unit, Karolinska University Hospital, Karolinska Institutet, Stockholm, Sweden; 10 Clinical Research Centre, Örebro University Hospital, Örebro, Sweden; Mahidol University, Thailand

## Abstract

**Background:**

The Roll Back Malaria strategy recommends a combination of interventions for malaria control. Zanzibar implemented artemisinin-based combination therapy (ACT) for uncomplicated malaria in late 2003 and long-lasting insecticidal nets (LLINs) from early 2006. ACT is provided free of charge to all malaria patients, while LLINs are distributed free to children under age 5 y (“under five”) and pregnant women. We investigated temporal trends in Plasmodium falciparum prevalence and malaria-related health parameters following the implementation of these two malaria control interventions in Zanzibar.

**Methods and Findings:**

Cross-sectional clinical and parasitological surveys in children under the age of 14 y were conducted in North A District in May 2003, 2005, and 2006. Survey data were analyzed in a logistic regression model and adjusted for complex sampling design and potential confounders. Records from all 13 public health facilities in North A District were analyzed for malaria-related outpatient visits and admissions. Mortality and demographic data were obtained from District Commissioner's Office. P. falciparum prevalence decreased in children under five between 2003 and 2006; using 2003 as the reference year, odds ratios (ORs) and 95% confidence intervals (CIs) were, for 2005, 0.55 (0.28–1.08), and for 2006, 0.03 (0.00–0.27); *p* for trend < 0.001. Between 2002 and 2005 crude under-five, infant (under age 1 y), and child (aged 1–4 y) mortality decreased by 52%, 33%, and 71%, respectively. Similarly, malaria-related admissions, blood transfusions, and malaria-attributed mortality decreased significantly by 77%, 67% and 75%, respectively, between 2002 and 2005 in children under five. Climatic conditions favorable for malaria transmission persisted throughout the observational period.

**Conclusions:**

Following deployment of ACT in Zanzibar 2003, malaria-associated morbidity and mortality decreased dramatically within two years. Additional distribution of LLINs in early 2006 resulted in a 10-fold reduction of malaria parasite prevalence. The results indicate that the Millennium Development Goals of reducing mortality in children under five and alleviating the burden of malaria are achievable in tropical Africa with high coverage of combined malaria control interventions.

## Abstract 2

Zanzibar has implemented artemisinin-based combination therapy for uncomplicated malaria, plus long-lasting insecticidal nets. Achuyt Bhattarai and colleagues found malaria morbidity and mortality decreased dramatically within two years.

## Editors' Summary

**Background.:**

Malaria kills about one million people every year, many of them young children living in sub-Saharan Africa. The parasite responsible for these deaths—Plasmodium falciparum—is transmitted to people when they are bitten (usually at night) by an infected mosquito. In the human body, the parasites reproduce in the liver before invading red blood cells. Here, they multiply again before bursting out and infecting more red blood cells as well as causing a high fever and sometimes damaging vital organs. The transmission cycle is completed when a mosquito bites an infected person and ingests parasites with its blood meal. To reduce the global burden of malaria, this cycle needs to be broken. This can be done in several ways. First, mosquitoes can be controlled with insecticides. Second, individuals can avoid mosquito bites by sleeping under insecticide-treated nets. Finally, antimalaria drugs can reduce the illness and death caused by the malaria parasite and can lessen the likelihood that a mosquito will pick up the parasite when it bites a person. The World Health Organization (WHO) currently recommends artemisinin-based combination therapies (ACTs) for malaria control. These contain a natural antimalarial compound from sweet wormwood and a synthetic drug. The use of insecticide-treated nets is also now being strongly promoted.

**Why Was This Study Done?:**

The Roll Back Malaria Partnership—a coordinated global approach to fighting malaria—recommends that the strategies described above be combined to control malaria. But, although the public-health impact of insecticidal nets (ITNs) has been investigated, the large-scale effect of ACT use and the combined ACT/ITN effect in a malaria-endemic area has not been studied. This information is needed to allow governments and international agencies to use their resources as effectively as possible to control malaria. In this study, the researchers have asked how the introduction of ACT, first alone and later combined with distribution of long-lasting insecticidal nets (LLINs), affected the malaria burden in Zanzibar, a malaria-endemic country. People with malaria have had free access to ACT in Zanzibar since late 2003; children under the age of 5 y and pregnant women have been given free LLINs since early 2006.

**What Did the Researchers Do and Find?:**

The researchers counted the parasites in the blood of a group of children under the age of 14 years in the North A District of Zanzibar in May 2003, 2005, and 2006 (the seasonal peaks for malaria in Zanzibar occur in March–May and October–December). They also looked in local health records for malaria-related outpatient visits and admissions between 2000 and 2005 and analyzed the overall death records for the region over the same period. Between 2003 (before the introduction of ACT) and 2005, the proportion of children under five with P. falciparum in their blood halved (a 2-fold decrease). It decreased another 10-fold between 2005 and 2006 after the distribution of LLINs to this age group. Deaths from all causes in children under five halved between 2002 and 2005, and malaria-related admissions and death attributed to malaria in 2005 in these children were one-fourth of those recorded in 2002. The climate in Zanzibar remained favorable for malaria transmission throughout this period.

**What Do These Findings Mean?:**

These findings show that malaria-associated illness (outpatient malaria diagnosis) decreased by 77% and overall deaths in children decreased to about half in Zanzibar within two years of the introduction of ACT. Free distribution of LLINs from early 2006 to children under five produced a further significant reduction in parasite prevalence in this age group and a smaller but also important reduction in parasite prevalence in older children. Because these results only show short-term trends in the malaria burden associated with the introduction of these control strategies, they need confirmation in longer studies. They also need confirmation in other countries because the malaria burden in Zanzibar could have fallen for reasons unrelated to ACT or LLINs, such as other changes in medical practice. Nevertheless, these results strongly suggest that ACTs together with the widespread use of LLINs could help achieve the goal of eliminating malaria as a public-health problem in sub-Saharan Africa, provided the poor countries in this region can sustain these control strategies over the long term.

**Additional Information.:**

Please access these Web sites via the online version of this summary at http://dx.doi.org/10.1371/journal.pmed.0040309.

- The MedlinePlus encyclopedia contains a page on malaria (in English and Spanish)
- Information is available from the World Health Organization on malaria (in English, Spanish, French, Russian, Arabic and Chinese)
- The US Centers for Disease Control and Prevention provide information on malaria and on insecticide-treated nets (in English and Spanish)
- Information is available from the Roll Back Malaria Partnership on its approach to global control of malaria, on malaria in Zanzibar, part of the United Republic of Tanzania, on artemisinin-based combination therapy, and on the use of insecticide-treated nets

## Introduction

The increased malaria-related morbidity and mortality, especially in children under the age of 5 y (“under five”), due to emerging resistance of Plasmodium falciparum to conventional antimalarial drugs calls for immediate actions to “Roll Back Malaria” in sub-Saharan Africa. This need has been clearly recognized in the Millennium Development Goals “to halt and begin to reverse malaria incidence” [1] as well as in the Abuja Declaration objective to halve malaria mortality in Africa by 2010 through implementation of combined control strategies [2].

In the year 2000, the overall treatment failure of chloroquine was found to be 60% in a 14-d efficacy trial; consequently the Zanzibar Ministry of Health and Social Welfare decided in November 2001 to change both first- and second-line treatment guidelines for uncomplicated malaria from chloroquine and sulfadoxine-pyrimethamine to artemisinin-based combination therapies (ACT) [3]. The ACT policy was implemented in September 2003, when Zanzibar became one of the first regions in sub-Saharan Africa to recommend routine use of ACT. This action was followed by strengthened vector control, culminating in a nation-wide distribution campaign of long-lasting insecticidal nets (LLINs) from early 2006.

Both ACT and vector control measures have independently proven to be efficacious malaria control strategies. Ecological studies have credited ACT with enhancing treatment efficacy, reducing malaria transmission, and possibly forestalling drug resistance in low-endemicity areas [4,5]. Moreover, specific African trials have indicated that the use of insecticide-treated nets (ITNs) or indoor residual spraying can reduce mortality of children under five in Africa [6–9]. This is, however, to our knowledge the first study to examine the public health impact of wide-scale deployment of ACTs alone and combined with ITNs through the general health structure/channels on malaria indices and general health parameters in an endemic area in sub-Saharan Africa.

## Methods

### Study Site

The study was conducted in North A District, Zanzibar, situated just off the coast of mainland Tanzania. The district is rural and has a population of about 85,000. Subsistence farming and fishing are the main occupations. Plasmodium falciparum is the predominant malaria species and Anopheles gambiae complex is considered the main vector. Malaria transmission is stable with seasonal peaks related to rainfall in March–May and October–December. Malaria transmission in the district prior to the interventions has been reported to be high, but specific entomological data are not available to allow a precise characterization of malaria transmission intensity. However, during the screening process of a major antimalarial drug trial conducted in 2002–2003, a P. falciparum prevalence exceeding 30% was observed in febrile children under five [10], suggesting that North A District had been a high transmission area prior to ACT implementation in September 2003.

North A District has one Primary Health Care Centre, which includes a hospital with inpatient and laboratory services, e.g., blood transfusion and malaria microscopy services. Basic medical treatment services without laboratory support are provided in 12 Primary Health Care Units located in different shehias (the smallest political administrative unit in Zanzibar). Drugs, including conventional and artemisinin monotherapies, are also available in private shops throughout the district.

### Malaria Control Interventions

**Figure 1:**
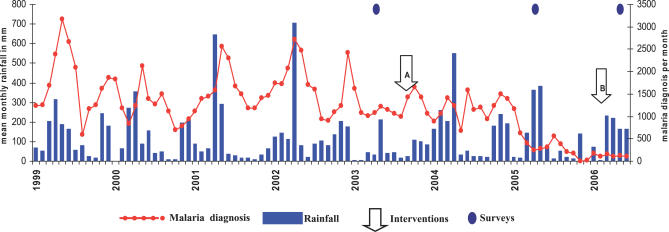
Malaria Interventions, Cross-Sectional Surveys, Monthly Rainfall, and Reported Clinical Malaria Diagnoses in Children under 5 Years of Age in North A District, Zanzibar (A) Start of the implementation of artemisinin-based combination therapy for treatment of uncomplicated malaria in September 2003. (B) Introduction of LLINs in February 2006. Promotion of ITNs started in January 2004; the use of conventional ITNs, however, remained low, until the introduction of LLINs. Outpatient data for 2006 are up to June.

Figure 1 illustrates time of implementation of the two malaria control interventions.

#### First intervention—ACT.

A loose combination of artesunate and amodiaquine (AS+AQ; from various suppliers with preapproval from WHO) and a fixed combination of artemether–lumefantrine (Coartem; Novartis, Basel, Switzerland), were implemented as first- and second-line treatment, respectively, for uncomplicated malaria in all public health facilities from September 2003. In a pre-implementation assessment of the new treatment policy, partly conducted in North A District 2002–2003, both AS+AQ and artemether–lumefantrine were highly efficacious with PCR-adjusted cure rates by day 28 above 90% [10]. Quinine remained the drug of choice for severe malaria and sulfadoxine-pyrimethamine for intermittent preventive treatment during pregnancy. From September 2003, chloroquine was withdrawn from all health facilities and replaced by free provision of ACT to all malaria patients. Total treatment courses of AS+AQ dispensed in North A 2004 and 2005 were 34,724 and 12,819, respectively. The supply of ACT has been uninterrupted, with no reports of AS+AQ being out of stock from any public health facility in the district during 2003–2006 (unpublished data). ACTs were purchased with support from African Development Bank and Global Fund to Fight AIDS, Tuberculosis and Malaria (GFATM).

#### Second intervention—vector control.

A policy to distribute conventional ITNs to the most vulnerable groups—children under five and pregnant women—free of charge through antenatal clinics or local shehia leaders was officially launched in 2004. However, ITN coverage and use remained low in North A District 2004 and 2005 due to limited number of ITNs distributed, 4,026 and 1,550, respectively. A mass campaign was therefore initiated early 2006, with distribution of 23,000 LLINs to the two most vulnerable groups in North A. This campaign was supported by GFATM and the US Agency for International Development.

### Cross-Sectional Surveys

Three cross-sectional surveys with the primary objective to determine P. falciparum prevalences were conducted in North A District between 2003 and 2006. A two-stage cluster sample technique was used. First shehias and then the households were randomly selected from the sampling frame obtained from the Office of Chief Government Statistician, Zanzibar. The sampling frame was updated before each survey.

The first exploratory survey, conducted in May 2003, included 625 households and provided baseline data prior to ACT and widespread ITN implementation. Sample size calculations for the follow-up surveys conducted in May 2005 and 2006 were based on the proportion of children under five with malaria parasitemia in 2003, about 9%, and an assumed relative error of 20%. The calculated number of households to be included was 490 after adjusting for a design effect of 2.

Trained interviewers visited all selected households. Interviews and blood sample collection were initiated upon written consent from head of each household and proxy consent from the mother or guardian of each child. Information was recorded using a structured questionnaire on recent febrile illness, mosquito net use, and care-seeking behavior from each individual present in the household at the time of the survey. We did not replace households in which residents were not present at time of survey, could not be located, or refused to participate.

Thick blood films were collected from all consenting participants, stained with 5% Giemsa for 30 min, and examined by experienced microscopists for presence and density of P. falciparum parasites. If fewer than ten parasites were detected per 200 white blood cells, examinations were extended to 500 white blood cells. Blood slides were considered negative if no asexual parasites were found in 200 high-power fields. High-density parasitemia was defined as presence of ≥ 5,000 parasites/μl [11]. Quality control was conducted for all positive slides and 10% of the negative slides [12].

### Health Facility Records

Malaria-related indicators, i.e., outpatient attendances, hospital admissions and blood transfusions, from all 13 public health facilities in North A District were obtained from the Health Management and Information System (HMIS) records of the Zanzibar Ministry of Health and Social Welfare. The existing HMIS records were about 90% complete for the period 2000–2004. Data were validated and missing information retrieved by retrospective review of source documents from all 13 health facilities. This confirmed the HMIS records and resolved missing or inconsistent data, which increased the completeness to nearly 100%. A database of malaria-related indicators was created on the basis of this retrospective review. Data from 2005 were abstracted on quarterly basis.

### Vital Statistics

Records of vital events, i.e., births and deaths, for the period 1998–2005 were obtained from the District Commissioner's Office (DCO) in North A. Annual crude mortalities of children under five were estimated from these data. Demographic estimates were obtained from Tanzania National Population and Housing Census 2002.

### Rainfall

Complete records of monthly rainfall during 1999–2005 were obtained from official registers of the Tanzania Metrological Agency of the Ministry of Communications and Transport. On Unguja island, rainfall is centrally measured in one weather station, situated 26 km (radially) from North A District. The mean annual rainfalls recorded in 2003, 2004, 2005, and 2006 were 702, 1,934, 1,231, and 1,214 mm, respectively. The corresponding mean seasonal rainfall (March–May) between 2003 and 2006 was 285, 786, 890, and 613 mm, respectively. During the post-ACT intervention period (2004–2006) the mean annual and seasonal rainfall was 8%–12% lower than the pre-ACT intervention period (2000–2002). However, the only year with a marked reduction in the mean annual and seasonal rainfalls was the year 2003 with two- to three-fold lower rainfall, as compared to both the preceding and subsequent 3 y.

### Data Processing and Analysis

Data were entered and validated using Microsoft Access and Excel. Statistical analyses for cross-sectional surveys, health facility records, vital statistics, and rainfall data were performed using Stata version 8. Analysis for the surveys was corrected for multi-stage sampling errors using the Rao-Scott second order correction [13]. A logistic regression model with robust standard errors (robust cluster) was used to adjust for the effect of age, sex, sleeping under a mosquito-net, and asset index on asexual P. falciparum prevalence and gametocyte carriage across the study years. Households were the primary sampling units in the surveys and were defined as clusters. Wald test was used to assess the fit of the model and interactions between covariates incorporated in the model. Odds ratios were adjusted for the complex sampling design and covariates listed above. Pearson correlation coefficients were calculated to assess the linear relationships between monthly rainfall and outpatient malaria diagnosis, and malaria-attributed deaths.

### Ethical Approval

Protocols for the household surveys were reviewed and approved by the Medical Research Coordinating Committee of the Tanzanian Commission on Science and Technology, the Zanzibar Health Research Council and the institutional review board of US Centers for Disease Control and Prevention.

## Results

### Cross-Sectional Surveys

The timings of the cross-sectional surveys in relation to start of each malaria control intervention and seasonal rainfalls are presented in Figure 1. The number of households enrolled and participant characteristics in the respective surveys are shown in Table 1. Over 95% of all participants agreed to both answer questionnaires and provide blood samples in the respective surveys.

**Table 1:**
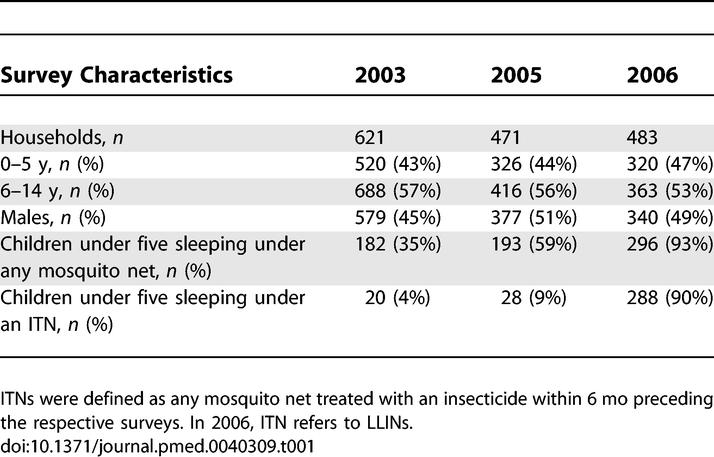
Number of Households Surveyed and Characteristics of Survey Participants

The parasite prevalences and odds ratios (ORs) of asexual P. falciparum parasitemia and gametocyte carriage at the time of cross-sectional surveys are shown in Table 2. Between 2003 and 2005 the parasite prevalence was reduced by about 50% in children under five. A further 10-fold decrease in P. falciparum prevalence was observed between 2005 and 2006, following mass distribution of LLINs specifically targeting this age group. Concomitant reductions of parasite prevalence were observed in children over the age of 5 y, although only by about 3-fold, between 2005 and 2006 (OR 0.41, 95% confidence interval [CI] 0.13–1.21), *p* = 0.08).

**Table 2:**
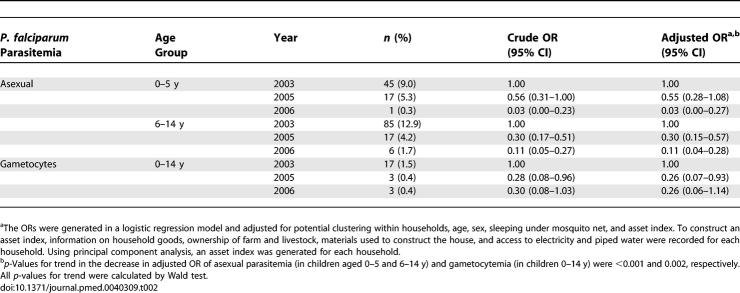
Parasite Prevalence and ORs of P. falciparum Asexual Parasitemia and Gametocytemia in Children 0–14 Years of Age in North A District, Zanzibar, in May 2003, 2005, and 2006

High-density parasitemia (≥5,000/μl) was found in 14 (2.7%) and 2 (0.6%) children under five in 2003 and 2005, respectively. No child carried high-density parasitemia in 2006.

Reported fever within 14 d prior to the survey was similar in 2003 and 2006 among children under five (2003, 13% [95% CI 11–17]; 2006, 12% [95% CI 9–16]), whereas care-seeking at public health facilities by recently febrile children under five increased significantly (2003 was reference year; 2005, OR 3.91 [95% CI 0.85–17.9]; 2006, OR 5.5 [95% CI 2.3–13.3]; *p*-value for trend < 0.001).

The proportions of children under five sleeping under effective ITNs were below 10% in both 2003 and 2005 (Table 1), whereas in 2006, 90% were reported sleeping under an LLIN on the night before survey**.**

### Health Facility Surveillance

All reported clinical outpatient malaria diagnoses in North A District between January 1999 and June 2006 among children under five are shown by month in Figure 1 and by year in Table 3. Between 2002 and 2005 the total number of out-patient malaria diagnoses decreased by 77%. The annual incidences of malaria diagnoses standardized per 1,000 children under five in North A District were 843, 786, and 233 in 2003, 2004, and 2005, respectively. The total number of children under five attending public health facilities for any cause during 1999 and 2005 remained relatively constant, ranging from 31,069 to 39,374 annually. Up to 2003 malaria accounted for about 50% of all outpatient diagnoses in this age group, whereas in 2005 this proportion had decreased to 13%.

**Table 3:**
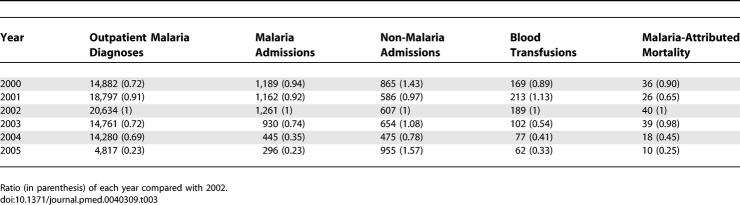
Outpatient Malaria Diagnoses, Hospital Admissions, Blood Transfusions, and Malaria-Attributed Deaths in North A District, Zanzibar, between 2000 and 2005

Malaria-related hospital admissions, non-malaria admissions, and blood transfusions in children under five between 2000 and 2005 are also shown in Table 3. From 2002 to 2005, malaria-related admissions, blood transfusions, and malaria-attributed mortality decreased by 77%, 67%, and 75%, respectively.

### Crude Mortality Data

A total of 23,200 live births and 1,032 deaths in children under five (49% females) were registered between January 1998 and December 2005. The annual mortality figures for children under five, children (1–4 y), and infants (0–1 y) are shown in Table 4. Between 2002 and 2005, crude under five, infant, and child mortality decreased by 52%, 33%, and 71%, respectively.

**Table 4:**
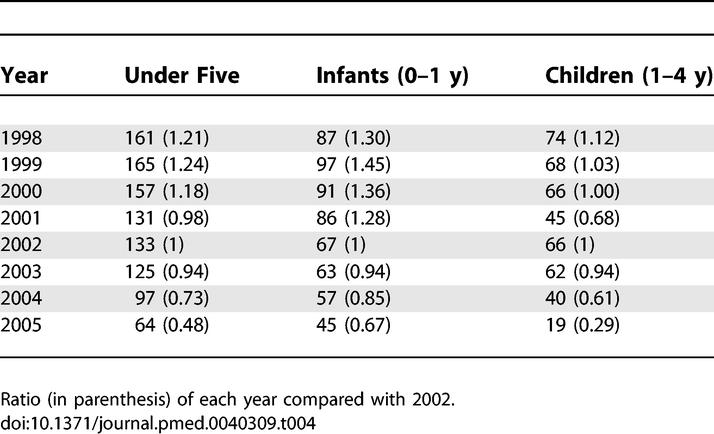
Mortality of Children under 5 Years of Age in North A District, Zanzibar between 1998 and 2005

### Relationships between Rainfall and Malaria Diagnosis and Deaths

In the pre-ACT intervention period (2000–2002), significant positive correlations were found between monthly rainfall and both outpatient malaria diagnoses (Pearson correlation coefficient [*r*_p_] = 0.59, *p* < 0.001) and malaria-attributed deaths (*r*_p_ = 0.75, *p* < 0.001), when data were adjusted to allow for a 1-mo lag between rainfall and malaria diagnoses and deaths. However, in the post-ACT intervention period (2003–2005), no significant correlations were found between monthly rainfall and outpatient malaria diagnosis (*r*_p_ = −0.05; *p* = 0.75) or malaria-attributed deaths (r_p_ = 0.23; *p* = 0.20).

## Discussion

Malaria burden in Zanzibar, as in most parts of sub-Saharan Africa, has remained high and in many areas even increased during the last 10–20 y, a major reason being rapid spread of resistance to commonly used monotherapies against malaria. This problem has necessitated urgent implementation of new and effective control strategies to “Roll Back Malaria.” Two main cornerstones in this effort are the introduction of ACTs for treatment of uncomplicated malaria and the promotion of ITN use. The targets for the implementation of these new strategies have been defined by the UN Millennium Development Goals [1] and the Abuja Declaration [2], to be achieved by the years 2015 and 2010, respectively.

### Deployment of ACTs

The ACTs were dispensed free of charge to all patients in the study area through public health facilities from September 2003 onwards. The ACT implementation and deployment was very rapid, effective, and with high coverage. Monitoring of drug supplies confirmed that ACTs were available throughout the study period in all 13 public health care settings in North A District. This outcome also indicates that estimates were adequate of the needed and thus deployed numbers of ACT treatments in the district. This result was accomplished despite an apparent two-fold increase in care seeking among children under the age of 5 y at public health facilities as observed in the cross-sectional surveys. We believe that the observed shift in treatment-seeking behavior at public facilities may be related to availability of free, effective ACTs. A previous study in Zanzibar showed that people's attitudes towards health seeking at public health facilities (biomedical practices) are negatively influenced by the distribution of ineffective antimalarial drugs [14].

High ACT coverage was rapidly achieved in malaria patients despite availability of other drugs in the private sector. This achievement was probably influenced both by comprehensive information to the public and health care staff and by the strong commitment of the Zanzibar government to rapidly ensure free coverage of the ACTs. Also, in North A District, as well as in Zanzibar generally, the entire population has relatively easy access to public health facilities, which are located within 5 km from any community and are served by good transport links. However, the absence of co-formulation or even of co-blistering of the two compounds in the first-line treatment, artesunate and amodiaquine, may have resulted in some degree of monotherapy with either compound.

### Mortality Impact

Our study provides the first, to our knowledge, observation of a reduction in mortality of children under five following introduction of ACTs solely in a stable malaria-endemic setting.

The highly significant reduction of 52% in crude under-five mortality according to vital statistics between 2002 and 2005 also highlights the importance of malaria as a major cause of death among children in malaria-endemic areas. The 71% reduction among children aged 1–4 y indicates that the relative contribution of malaria to crude mortality is particularly important in this age group. Major reductions in crude under-five mortality has also been observed in previous randomized intervention studies with ITNs [6,7] and community-based malaria treatment [15,16], but the reduction rates (between 25% and 40%) have been less pronounced than those in our study in Zanzibar.

We believe our findings are valid and represent a true picture of the effects of ACT deployment in North A District, Zanzibar. No other major political, socioeconomic, or health-care change with the potential to halve mortality in children under five occurred in Zanzibar after 2002. This includes Expanded Programme on Immunization coverage, which remained constantly above 80% in the district during 1999–2005. Furthermore, there was no significant change in rainfall that may have contributed to the observed reduction in malaria transmission. Indeed, the only year with reduced rainfall with potential influence on vector capacity occurred before the introduction of ACTs—in 2003. Increased use of ITNs may also represent a potential confounding factor in our study. However, the ITN use was below 10% during 2004 and 2005 as reported and observed during the cross-sectional surveys. A significant improvement in ITN coverage was only achieved in 2006 after the introduction of LLINs (see further below) and only affected the 2006 cross-sectional results.

We chose 2002 as reference year in our analyses of health facility surveillance and under-five mortality, because 2002 represents the last complete year before ACT introduction in September 2003. Routinely collected mortality statistics may underestimate the true values. However, such data have been shown to provide valid mortality trends [17,18].

### Morbidity Impact

A significant reduction was found with regard to hospitalization of malaria patients and incidence of blood transfusions, which may be considered proxy indicators of severe malaria. The reduction of severe malaria showing a similar pattern thus supports the under-five mortality trends. This health impact probably represents effects of improved case management of uncomplicated malaria with ACT, thus preventing the development of severe manifestations of the disease. The decrease in malaria morbidity (and mortality) at health facilities between 2003 and 2005 confirms the therapeutic efficacy of ACT [10], but the reduction in outpatient malaria diagnoses may also reflect some transmission blocking effect of artemisinin derivatives through its gametocytocidal activity. Reduction in transmission potential has been suggested after the introduction of artemisinin derivatives (before vector control) for routine treatment in a low and seasonal malaria transmission setting in Thailand [4].

Data obtained from routine health facility records have inherent potential pitfalls and need to be interpreted cautiously. However, the fact that they all show the same downward trend after improved coverage of malaria prevention and treatment interventions, and with no change in the climatic conditions that are favorable for malaria transmission, supports the plausible conclusion that enhanced malaria control interventions contributed to the observed public health benefits.

### Deployment of ITNs

The deployment of LLINs in early 2006 provided a high coverage, i.e., over 90% reported use in children under five in the cross-sectional survey in May 2006. Importantly, this high mosquito-net use was observed after strong government commitment and after free LLIN distribution to children under five and pregnant women.

The most significant decrease in prevalence of asymptomatic parasitemia was achieved in 2006, when LLINs were widely used by the children under five, whereas the major impact on the under-five mortality was achieved earlier with ACT use only.

Strengthened vector control and the use of ACT also resulted in marked and sustained malaria control in South Africa [5]. The similar public health benefits observed in North A supports the concomitant use of vector control and ACT for malaria control. However, it should be emphasized that our study captures short-term trends in malaria control in North A, which may be too short to generalize long-term trends in the burden of malaria. Sustained coverage and use of LLINs by vulnerable groups is yet to be demonstrated, especially under declining malaria endemicity and if the free LLIN distribution scheme were to be changed.

### Conclusions

The declining under-five mortality, malaria morbidity, and malaria prevalence observed in our study is the first comprehensive evidence supporting the major public health benefits of ACT and ITNs in a stable endemic malaria transmission setting in sub-Saharan Africa.

The findings suggest that ACTs with high coverage of ITN use may potentially even eliminate malaria as a public health problem in highly endemic areas of sub-Saharan Africa. High community uptake of the two interventions is probably required but indeed achievable if, as in our study, they are easily available free of charge.

The UN Millennium Development Goals to alleviate malaria as a major public health problem and substantially reduce the under-five mortality in sub-Saharan Africa are thus achievable even in settings with historically intense malaria transmission. The sustainability of these efforts as well as surveillance to prevent resurgence of malaria represent key research and programmatic follow-up issues of malaria control in Africa.

## Abbreviations

ACT: artemisinin-based combination therapy
AQ: amodiaquine
AS: artesunate
CI: confidence interval
GFATM: Global Fund to Fight AIDS, Tuberculosis and Malaria
HMIS: Health Management and Information System
ITN: insecticide-treated net
LLIN: long-lasting insecticidal net
OR: odds ratio
